# A reinforcement learning‐based hybrid modeling framework for bioprocess kinetics identification

**DOI:** 10.1002/bit.28262

**Published:** 2022-10-26

**Authors:** Max R. Mowbray, Chufan Wu, Alexander W. Rogers, Ehecatl A. Del Rio‐Chanona, Dongda Zhang

**Affiliations:** ^1^ Department of Chemical Engineering, Centre for Process Integration University of Manchester Manchester UK; ^2^ Centre for Process Systems Engineering Imperial College London London UK

**Keywords:** historical‐dependent kinetics, hybrid modeling, model structure identification, reinforcement learning, time‐varying parameter estimation

## Abstract

Constructing predictive models to simulate complex bioprocess dynamics, particularly time‐varying (i.e., parameters varying over time) and history‐dependent (i.e., current kinetics dependent on historical culture conditions) behavior, has been a longstanding research challenge. Current advances in hybrid modeling offer a solution to this by integrating kinetic models with data‐driven techniques. This article proposes a novel two‐step framework: first (i) speculate and combine several possible kinetic model structures sourced from process and phenomenological knowledge, then (ii) identify the most likely kinetic model structure and its parameter values using model‐free Reinforcement Learning (RL). Specifically, Step 1 collates feasible history‐dependent model structures, then Step 2 uses RL to simultaneously identify the correct model structure and the time‐varying parameter trajectories. To demonstrate the performance of this framework, a range of in‐silico case studies were carried out. The results show that the proposed framework can efficiently construct high‐fidelity models to quantify both time‐varying and history‐dependent kinetic behaviors while minimizing the risks of over‐parametrization and over‐fitting. Finally, the primary advantages of the proposed framework and its limitation were thoroughly discussed in comparison to other existing hybrid modeling and model structure identification techniques, highlighting the potential of this framework for general bioprocess modeling.

## 1 INTRODUCTION

Mathematical modeling provides a significant contribution to the understanding and design of biochemical reaction processes. A properly validated model can predict bioprocess dynamics under different operating conditions such as light intensity for algal and cyanobacterial photo‐production systems (Del rio‐Chanona et al., 2017), and temperature (Rivera et al., 2007) and pH (Smolders et al., 1994) for general fermentation. It can estimate the effects of key process operating conditions on biomass growth and product synthesis without the need of running extra experiments (Sadino‐Riquelme et al., 2020). Different predictive models have been proposed to simulate complex biological processes, such as kinetic models (Gadhe et al., 2014; Kyriakopoulos et al., 2018), machine learning‐based data‐driven models (Adeniyi et al., 2021; Bradford et al., 2018; Dineshkumar et al., 2015; Svendsen et al., 2020), and hybrid models (i.e., integration of the former two). Each paradigm comes with its own modeling nuances.

For example, within the area of kinetic modeling, it is common for models to be over‐parameterized (i.e., characterized by many kinetic parameters and multiple structures) in an effort to capture the complex dynamics expressed within the data. However, this often leads to issues related to practical identifiability and nonlinear model parameter estimation, resulting in high parameter uncertainty, which hinders the identification of physical knowledge derived from certainty in kinetic parameter estimation. In reality, issues associated with parameter identifiability often come from changes in enzymatic activity as a result of the metabolic regulation system (Aboulmouna et al., 2020). Similarly, there is potential within data‐driven approaches to overfit experimental training data used in model construction. This leads to poor model generalization when making predictions on previously unseen data. Both phenomena have the potential to arise in hybrid models too.

Among them, hybrid models are considered the new trend for bioprocess predictive modeling and have been applied to several recent studies. These models either use a data‐driven model to rectify the mismatch between a kinetic model and available data, or use a data‐driven model to estimate kinetic model parameters directly. For example, Zhang et al. (2019) applied an artificial neural network (ANN) to identify optimal control actions and predict future process behaviors for bioprocess online simulation and optimization, and demonstrated that the hybrid modeling framework could resolve practical issues such as lack of physical knowledge of processes and low quality and quantity of collected data. Vega‐Ramon et al. (2021) used Gaussian process regression models to frequently update parameters of a kinetic model for robust modeling of a yeast astaxanthin production process and demonstrated that the hybrid model could reduce simulation errors by over 60% and decrease uncertainty by over 50% compared with the kinetic model, which is particularly robust to uncertainty propagation.

Nonetheless, all modeling methods have their limits. Kinetic models usually require several time‐consuming trial‐and‐error iterations to identify the “correct” model structure (Svendsen et al., 2020; Zhang et al., 2020). Data‐driven models use a lumped parameter description to capture underlying process dynamics without physical knowledge but strongly rely on the quantity and quality of data collected from experiments (Sansana et al., 2021). Although a hybrid model can effectively minimize model fitting errors through the combination of data‐driven and kinetic models, it still introduces a severe risk of over‐parameterizing the kinetic model part or overfitting the data‐driven model part, thus leading to unreliable predictions and high model uncertainty. As a result, identifying an appropriate kinetic model structure to represent underlying process mechanisms and reducing dependence on using an over‐fitted data‐driven model is critical to the success of a hybrid model.

However, finding a reliable kinetic model structure is not trivial. For instance, although classic kinetic models such as the Monod model, the Contois model, and the Haldane model have been used to describe a range of bioprocesses (Annuar et al., 2008; Hamitouche et al., 2012; Krishnan et al., 2017), they are often incapable of simulating the entire process dynamics alone. For example, Chackoshian Khorasani et al. (2013) had to build a model combining two classic kinetic models to describe behaviors of mazut biodegradation at different conditions (e.g. change of mazut concentration, pH, and electrical potential). In addition, Zhang et al. (2015) proposed a piecewise model to simulate hydrogen production via *R. palustris* photo‐fermentation by switching between different kinetic models under different growth phases and changes in biochemistry; a challenge that few models can accomplish by themselves.

Fundamentally, the heterogeneity of a microbial culture makes it challenging to use a single kinetic model to simulate the entire bioprocess dynamics; each cell behaves differently depending on its individual lifecycle stage and micro‐environmental history (Sunya et al., 2013). Therefore, the current bioprocess kinetics are determined by the present culture conditions and the process history, a significantly different concept from a typical chemical reaction system (Casadesús & D'Ari, 2002). This historical dependence arises incidentally due to stochastic effects on the controlling mechanisms or metabolic stores; but is also systematically introduced by intracellular metabolic regulation mechanisms (Wolf et al., 2008). As a result, bioprocess kinetics can continuously evolve during an ongoing process, meaning that either its model structure or its parameters (or both) will change with respect to time and history (Vega‐Ramon et al., 2021; Wolf et al., 2008). However, it is extremely challenging to perform time‐varying parameter estimation or history‐dependence model structure identification (Liu, 2020; Van Impe, 1996). Despite their practical importance, little effort has been focused on these areas. Although there are a few recent attempts to tackle these issues for specific case studies (Cabaneros Lopez et al., 2021; Zhang et al., 2020), the applicability of these methods for general bioprocesses remains unexplored.

Therefore, in this study, we propose a novel framework to resolve these challenges by integrating physics‐informed hybrid modeling and reinforcement learning. The main idea of this framework is first to construct a hybrid model consisting of history‐dependent kinetic model structures and time‐varying parameters. Then, reinforcement learning is employed to simultaneously identify the most probable model structure for a set of process measurements and estimate the model's parameters. Specifically, model‐free reinforcement learning is chosen as it uses a trial‐and‐error approach to learn an optimal decision policy for sequential decision‐making problems. To demonstrate the framework's efficiency, three scenarios are explored via in‐silico experiments. Each scenario is comprised of two case studies, reflecting two different levels of complexity of the underlying process. The structure of this paper is as follows: Section 2 will first define the three scenarios; Section 3 will explain how the model structure identification and parameter estimation problem is formulated in terms of an RL control problem; then Section 4 will discuss the results; finally, Section 5 will conclude.

## 2 PROBLEM STATEMENT

To evaluate the competence of the RL‐based hybrid modeling framework, we considered three scenarios commonly observed in biochemical processes. To ensure generality, computational experiments were used to generate several in silico experimental datasets. Each in silico experiment simulated 168 h of fermentation to reflect a typical fermentation process. Biomass, substrate, and product concentration were measured every 2 h in Scenarios 1 and 2, but every 28 h in Scenario 3 (i.e., scarce data scenario). Finally, 5% additive white noise is assumed to exist in the measured data.

### 2.1 Introduction to Scenario 1: Combinatorial kinetic systems

Scenario 1 explores the framework's ability to capture the kinetics of a biological process that consists of two types of kinetic behavior. Several fermentation processes such as Chackoshian Khorasani et al. (2013); Hidayat et al. (2021); Wang et al. (2016) have been found to fall within this category. Equations (1a)–(1c) reflect the ground‐truth kinetics for Scenario 1 and are used to generate the in silico experimental data. The ground‐truth model incorporates the Monod model (first term on the right‐hand side) and the Contois model (second term on the right‐hand side). Parameters for the ground‐truth model are listed in Table 1. In this study, two in silico datasets were generated with a 5% Gaussian measurement noise from an initial biomass concentration of 0.15 g L^−1^, substrate concentration of 7.5 g L^−1^ and product concentration of 0.0 mg L^−1^.

(1a) $$\frac{\mathrm{dX}}{\mathrm{dt}}=\left(\mu_{\mathrm{mm}}∙\frac{S}{K_{\mathrm{sm}}+S}+\mu_{mc}∙\frac{S}{K_{\mathrm{sc}}∙X+S}\right)∙X$$

(1b) $$\frac{\mathrm{dS}}{\mathrm{dt}}=-Y_{S}∙\left(\mu_{\mathrm{mm}}∙\frac{S}{K_{\mathrm{sm}}+S}+\mu_{\mathrm{mc}}∙\frac{S}{K_{\mathrm{sc}}∙X+S}\right)∙X$$

(1c) $$\frac{\mathrm{dP}}{\mathrm{dt}}=Y_{P}∙\left(\mu_{\mathrm{mm}}∙\frac{S}{K_{\mathrm{sm}}+S}+\mu_{\mathrm{mc}}∙\frac{S}{K_{\mathrm{sc}}∙X+S}\right)∙X$$

where $X\in R^{+}$, $S\in R^{+}$, and $P\in R^{+}$ are the concentration of biomass, substrate, and product, respectively; $\mu_{\mathrm{mm}}\in R^{+}$ and $\mu_{\mathrm{mc}}\in R^{+}$ are the maximum specific growth rate of Monod and Contois model, respectively; $K_{\mathrm{sm}}\in R^{+}$ and $K_{\mathrm{sc}}\in R^{+}$ are the substrate saturation constant of Monod and Contois model, respectively; $Y_{S}\in R^{+}$ is the substrate yield coefficient; and, $Y_{P}\in R^{+}$ is the product yield coefficient.

**Table 1:** Ground‐truth kinetic model parameters for Scenario 1 and 2

	Kinetic parameters	Value	Units
Scenario 1	$\mu_{\mathrm{mm}}$	0.045	$h^{-1}$
	$\mu_{\mathrm{mc}}$	0.140	$h^{-1}$
	$K_{\mathrm{sm}}$	8.0	${\mathrm{g L}}^{-1}$
	$K_{\mathrm{sc}}$	15.0	${\mathrm{g L}}^{-1}$
	$Y_{s}$	0.6	${\mathrm{g g}}^{-1}$
	$Y_{p}$	0.3	${\mathrm{mg g}}^{-1}$
Scenario 2	$\mu_{\mathrm{mc},t}$	0.1	$h^{-1}$
	$\mu_{\mathrm{mc},t-1}$	0.05	$h^{-1}$
	$K_{\mathrm{sc}}$	5.0	${\mathrm{g L}}^{-1}$
	$Y_{s}$	0.8	${\mathrm{g g}}^{-1}$
	$Y_{p}$	0.35	${\mathrm{mg g}}^{-1}$
	$\mu_{\mathrm{mc},t}$	0.1	$h^{-1}$

Scenario 1 is decomposed into two case studies. In the first case study, the proposed kinetic model structure was identical to the ground‐truth structure described by Equations (1a)–(1c). Therefore, the first case study aims to test whether the framework can recover the ground‐truth parameters from the experimental data. In the second case study, a different kinetic model structure was proposed, as shown in Equations (1d)–(1f). This is more realistic as in most cases it is not possible to identify the correct model structure a‐priori. A common approach is to list all possible model structures for investigation. Therefore, in addition to the Monod and Contois models which correctly describe the ground‐truth, the Haldane model is also added (third term on the right‐hand side), where $K_{i}\in R$ is a substrate inhibition constant. From the same experimental data, the second case study aims to test whether the framework can accurately recover the ground‐truth parameters and identify the correct model structure.

(1d) $$\frac{\mathrm{dX}}{\mathrm{dt}}=(\mu_{\mathrm{mm}}∙\frac{S}{K_{\mathrm{sm}}+S}+\mu_{mc}∙\frac{S}{K_{\mathrm{sc}}∙X+S}+\mu_{\mathrm{mh}}\frac{S}{K_{\mathrm{sh}}+S+\frac{S^{2}}{K_{i}}})∙X$$

(1e) $$\frac{\mathrm{dS}}{\mathrm{dt}}=-Y_{s}∙(\mu_{\mathrm{mm}}∙\frac{S}{K_{\mathrm{sm}}+S}+\mu_{\mathrm{mc}}∙\frac{S}{K_{\mathrm{sc}}∙X+S}+\mu_{\mathrm{mh}}\frac{S}{K_{\mathrm{sh}}+S+\frac{S^{2}}{K_{i}}})∙X$$

(1f) $$\frac{\mathrm{dP}}{\mathrm{dt}}=Y_{p}∙(\mu_{\mathrm{mm}}∙\frac{S}{K_{\mathrm{sm}}+S}+\mu_{mc}∙\frac{S}{K_{\mathrm{sc}}∙X+S}+\mu_{\mathrm{mh}}\frac{S}{K_{\mathrm{sh}}+S+\frac{S^{2}}{K_{i}}})∙X.$$

### 2.2 Introduction to Scenario 2: History‐dependent kinetic systems

Scenario 2 aims to investigate the framework's efficiency in modeling bioprocesses with history‐dependent behavior. This represents a range of bioprocesses such as those studied in (Casadesús & D'Ari, 2002; Wolf et al., 2008). Here, the in‐silico experiments were simulated using a history‐dependent ground‐truth model, shown in Equations (2a)‐(2c). The ground‐truth parameters for this model are listed in Table 1. In this model, biomass growth is assumed to be dependent on both the current (first term on the right‐hand side) and the previous (second term on the right‐hand side) states due to a regulatory feedback mechanism. As the effects of the current and previous states may not have an equal weight on biomass growth, their respective specific growth rates (i.e., $\mu_{\mathrm{mc},t}$ and $\mu_{\mathrm{mc},t-1}$ in the case of the Contois model) are assumed to be different (Fouchard et al., 2009). It is worth mentioning that although other kinetic parameters can also vary between different terms (current or past), their value remains constant in this study. A more complicated scenario will be discussed in the next section. Again, two datasets were generated, with an initial biomass concentration of 0.15 g L^−1^, substrate concentration of 7.5 g L^−1^ and product concentration of 0.0 mg L^−1^.

(2a) $$\frac{dX_{t}}{\mathrm{dt}}=\mu_{t}∙X_{t}=\left(\mu_{\mathrm{mc},t}∙\frac{S_{t}}{K_{\mathrm{sc}}∙X_{t}+S_{t}}+\mu_{\mathrm{mc},t-1}∙\frac{S_{t-1}}{K_{\mathrm{sc}}∙X_{t-1}+S_{t-1}}\right)∙X_{t}$$

(2b) $$\frac{dS_{t}}{\mathrm{dt}}=-Y_{S}∙\left(\mu_{\mathrm{mc},t}∙\frac{S_{t}}{K_{\mathrm{sc}}∙X_{t}+S_{t}}+\mu_{\mathrm{mc},t-1}∙\frac{S_{t-1}}{K_{\mathrm{sc}}∙X_{t-1}+S_{t-1}}\right)∙X_{t}$$

(2c) $$\frac{dP_{t}}{\mathrm{dt}}=Y_{P}∙\left(\mu_{\mathrm{mc},t}∙\frac{S_{t}}{K_{\mathrm{sc}}∙X_{t}+S_{t}}+\mu_{\mathrm{mc},t-1}∙\frac{S_{t-1}}{K_{\mathrm{sc}}∙X_{t-1}+S_{t-1}}\right)∙X_{t}$$

where $X_{t}\in R^{+}$, $S_{t}\in R^{+}$, and $P_{t}\in R^{+}$ are the current concentration of biomass, substrate, and product, respectively; and, $X_{t-1}\in R^{+}$, $S_{t-1}\in R^{+}$, and $P_{t-1}\in R^{+}$ are the ‘memory’ of the state concentration (i.e., the previous state concentration).

As with Scenario 1, Scenario 2 is also decomposed into two case studies: first proposing the correct model structure (a history‐dependent Contois model) and the second adding another kinetic term (a history‐dependent Monod model) as shown in Equations (2d)–(2f).

(2d) $$\frac{\mathrm{dX}}{\mathrm{dt}}=(\mu_{mc,t}∙\frac{S_{t}}{K_{\mathrm{sc}}∙X_{t}+S_{t}}+\mu_{\mathrm{mc},t-1}∙\frac{S_{t-1}}{K_{\mathrm{sc}}∙X_{t-1}+S_{t-1}}+\mu_{\mathrm{mm},t}∙\frac{S_{t}}{K_{\mathrm{sm}}+S_{t}}+\mu_{\mathrm{mm},t-1}∙\frac{S_{t-1}}{K_{\mathrm{sm}}+S_{t-1}})∙X_{t}$$

(2e) $$\frac{\mathrm{dS}}{\mathrm{dt}}=-Y_{s}∙\left(\mu_{\mathrm{mc},t}∙\frac{S_{t}}{K_{\mathrm{sc}}∙X_{t}+S_{t}}+\mu_{\mathrm{mc},t-1}∙\frac{S_{t-1}}{K_{\mathrm{sc}}∙X_{t-1}+S_{t-1}}+\mu_{\mathrm{mm},t}∙\frac{S_{t}}{K_{\mathrm{sm}}+S_{t}}+\mu_{\mathrm{mm},t-1}∙\frac{S_{t-1}}{K_{\mathrm{sm}}+S_{t-1}}\right)∙X_{t}$$

(2f) $$\frac{\mathrm{dP}}{\mathrm{dt}}=Y_{p}∙(\mu_{\mathrm{mc},t}∙\frac{S_{t}}{K_{\mathrm{sc}}∙X_{t}+S_{t}}+\mu_{\mathrm{mc},t-1}∙\frac{S_{t-1}}{K_{\mathrm{sc}}∙X_{t-1}+S_{t-1}}+\mu_{\mathrm{mm},t}∙\frac{S_{t}}{K_{\mathrm{sm}}+S_{t}}+\mu_{\mathrm{mm},t-1}∙\frac{S_{t-1}}{K_{\mathrm{sm}}+S_{t-1}})∙X_{t}.$$

### 2.3 Introduction to Scenario 3: Time‐varying kinetic systems

Scenario 3 aims to evaluate the performance of the framework for bioprocesses with time‐varying kinetic parameters. Kinetic parameters in this scenario change at each time step to reflect changes in intracellular metabolic reactions activity. Many bioprocesses have been found to belong to this scenario (O'Brien et al., 2021; Pinto et al., 2019; Wang et al., 2010). This is the main area where hybrid modeling thrives in outperforming pure kinetic and data‐driven models. The hybrid model allows for changes of parameters to improve kinetic model accuracy without imposing a more complex kinetic model structure. However, the retention of the mechanistic model structure constrains the flexibility of the data‐driven model, which can often overfit the available data when predictions are made at each time index.

Once again, two case studies were designed. The first case study tests whether the framework can recover the time‐varying ground‐truth parameters correctly, now that there is a high degree of freedom for parameter estimation (i.e. all of the parameters can vary at each time step). Scenario 3 uses the time‐varying ground‐truth model shown as Equations (3a)–(3c) to generate the in‐silico experiments. Both case studies follow the same ground‐truth model structure but differ in how many parameters are time‐varying. For the first case study, most of the model parameters are time‐varying where their values and ranges are summarized in Table 2. However, in reality not all parameters change significantly over time. Therefore, for the second case study, only $\mu_{\mathrm{mc}}$ is changed at each time step where the parameter values and ranges are listed in Table 2. For both case studies in Scenario 3, two data sets were generated, with initial biomass concentration of 0.15 g L^−1^, substrate concentration of 7.5 g L^−1^ and product concentration of 0.0 mg L^−1^.

(3a) $$\frac{\mathrm{dX}}{\mathrm{dt}}=\left(\mu_{m}∙\frac{S}{K_{s}∙X+S}\right)∙X$$

(3b) $$\frac{\mathrm{dS}}{\mathrm{dt}}=-Y_{s}∙\left(\mu_{m}∙\frac{S}{K_{s}∙X+S}\right)∙X$$

(3c) $$\frac{\mathrm{dP}}{\mathrm{dt}}=Y_{P}∙\left(\mu_{m}∙\frac{S}{K_{s}∙X+S}\right)∙X.$$

**Table 2:** Ground‐truth kinetic model parameters for Scenario 3

Kinetic parameters	Parameter values for each time interval						Units
Case Study 1	$t_{0}$	$t_{1}$	$t_{2}$	$t_{3}$	$t_{4}$	$t_{5}$	
$\mu_{m}$	0.1	0.2	0.3	0.27	0.23	0.18	$h^{-1}$
$K_{s}$	6.0	6.0	6.0	6.0	6.0	6.0	${\mathrm{g L}}^{-1}$
$Y_{s}$	0.6	0.65	0.75	0.7	0.67	0.63	${\mathrm{g g}}^{-1}$
$Y_{p}$	0.1	0.12	0.16	0.23	0.35	0.6	${\mathrm{mg g}}^{-1}$
Case Study 2	$t_{0}$	$t_{1}$	$t_{2}$	$t_{3}$	$t_{4}$	$t_{5}$	
$\mu_{m}$	0.01	0.04	0.06	0.03	0.02	0.01	$h^{-1}$
$K_{s}$	8.0	8.0	8.0	8.0	8.0	8.0	${\mathrm{g L}}^{-1}$
$Y_{s}$	0.65	0.65	0.65	0.65	0.65	0.65	${\mathrm{g g}}^{-1}$
$Y_{p}$	0.3	0.3	0.3	0.3	0.3	0.3	${\mathrm{mg g}}^{-1}$

*Note*: Parameter values are fixed between time interval *t*_*i*_ and the next *t*_*i*+1_. Time‐varying parameter values change from one time step to the next whilst fixed parameters are the same.

## 3 METHODOLOGY

The reinforcement learning (RL) based hybrid modeling framework includes two steps: (i) speculate a list of possible kinetic model structures for the underlying process through domain‐specific phenomenological knowledge; and (ii) learn an RL policy to identify the most likely model structure and simultaneously estimate its associated model parameters via the principle of Frequentist estimation. As the first step has been described in Section 2, this section will mainly focus on the second step.

### 3.1 Framework formulation

**Figure 1:**
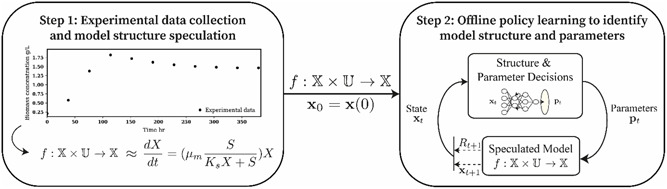
RL‐based two‐step hybrid modeling framework.

Formally, RL problems are modeled as a Markov Decision Process (MDP), which provides a mathematical framework for decision‐making within a stochastic process. In this study, we assume the problem of synchronously estimating model parameters, $p_{t}$, and identifying model structure can be modeled as an MDP. There is a set of measurable state variables $x_{t}\in X\subseteq R^{n_{x}}$, which characterize system evolution over a finite discrete time horizon, t∈{0,…,T}. To formulate the parameter estimation problem as a control problem, the kinetic model parameters $p_{t}\in U\subset R^{n_{p}}$, become control inputs to the kinetic models proposed in Section 2. Between each discrete time step the kinetic model parameters, $p_{t}$, take on a fixed value set by the control policy, $p_{t}=\pi (x_{t}^{m};\;\theta )$, which is a nonlinear function of the state variables, $x_{t}^{m}$, at the current time index. The state of the system in response to the control action at the next time step is then described by the kinetic model $x_{t+1}^{m}=f(x_{t}^{m},p_{t})$. The quality of the control action at each time is ranked by a reward function, $R:X\times U\times X\to R_{t+1}\in R$, based on how close the response is to the desired response. In other words, how close the model's prediction $x_{t+1}^{m}$ is to the experimentally measured state at that time $x_{t+1}^{d}$ – the smaller the error the larger the reward. The mathematical formulation of the overall RL‐based hybrid modeling framework is expressed as Equations (4a)–(4e), and its concept is illustrated in Figure 1.

(4a) $$\text{ma}x_{\pi (∙)}J=E_{\pi }\left[\sum_{t=0}^{T-1}R({x^{m}}_{t},p_{t},{x^{m}}_{t+1})\right]$$

(4b) $$s.t.{x^{m}}_{0}=x^{\exp}(0)$$

(4c) $${x^{m}}_{t+1}=f({x^{m}}_{t},p_{t})$$

(4d) $$p_{t}\in U.$$

(4e) $$p_{t}=\pi ({x^{m}}_{t})$$

From Equation (4a), the framework aims to find an optimal policy π, to maximize the expected criterion function J, which is the expected sum of the rewards over the time horizon subject to a given set of constraints including initial conditions (Equation (4b)), process dynamics (Equation (4c)) and hard constraints on the control inputs (i.e., physical bound of kinetic parameters if known, Equation (4d)). As usual, the definition of the objective function is flexible. To promote the sparsity of the predictions from the policy, one could directly penalize the zero norm of the kinetic parameters or, for example, regularize the change in the kinetic parameters from one time index to the next. This was not deemed necessary in the results presented.

The optimal parameters, $p_{t}^{*}$, returned by the approximately optimal control policy, $p_{t}^{*}=\pi^{*}(x_{t}^{m};\;\theta )$, constitute the final estimated kinetic model parameters. Since $p_{t}^{*}$ is different for each time step, all parameters will, in principle vary with time. To prevent overfitting, if a parameter is known to be constant over the time course (e.g., a‐priori knowledge), a penalty can be added in Equation (4a) to penalize its change over time steps. However, this was not required in this study. Alternatively, if this information is not known beforehand, once the policy is trained, if the values of a parameter, $p_{i}^{*}$, are found to be sufficiently similar from one‐time step, an average will be taken afterward so that the framework still returns a fixed parameter value to the hybrid model. This examination is carried out during validation instead of policy training.

### 3.2 Framework implementation

For most fermentation studies, the data is comprised of measurements of the concentrations of major state variables, such as biomass, substrate, and product. The initial conditions, possible kinetic model structures, and the hard constraints on the parameters in Equations (4b)–(4d) are informed by domain‐specific knowledge.

In general, optimization approaches to model structure identification and parameter estimation, use existing data and the likelihood function to provide a ranking over model parameters and structures (Wieland et al., 2021). In this study, we follow a similar line and propose to equate the objective function (i.e., Equation (4a)) to the likelihood function (or e.g., a weighted variant). By assuming that the residuals between the model prediction, $x_{i}^{m}$, and data, $x_{i}^{d}$, are zero mean Gaussian with constant variance, we can form a weighted least‐squares expression as Equation (5) that minimizes errors between the model prediction and experimental data:

(5) $$R\left(x_{t},p_{t},x_{t+1}\right)=-{\left(x_{t+1}^{m}-x_{t+1}^{d}\right)}^{T}W(x_{t+1}^{m}-x_{t+1}^{d})$$

where $x_{t+1}^{m}\in R^{n_{x}}$ and $x_{t+1}^{d}\in R^{n_{x}}$ are the model prediction and the experimental data at the next time index, t+1, respectively; and, $W=\mathrm{diag}\left(w\right)\in R^{n_{x}\times n_{x}}$, where $w=\left[w_{1},\text{…},w_{n_{x}}\right]$, is a weighting. In this study, we define w=1/max(A), where max(A) returns a column‐wise maximum. In practice, we additionally exponentiate Equation (6) to aid the efficiency of gradient‐based RL policy training (Mowbray et al., 2021). This can help with practical policy convergence but is not necessary in the context of the framework. However, it does have the added benefit of bounding $R\left(x_{t},p_{t},x_{t+1}\right)=[0,1]$, which enables us to analyze the results of the framework with respect to some theoretical maximum objective performance. Bounding the rewards is also known to provide stability in learning an approximation of the action‐value function (Mnih et al., 2015). The general objective function (Equation (4a)) can be written as:

(6) $$J=E_{\pi }\left[\sum_{t=0}^{T-1}-{\left(x_{t+1}^{m}-x_{t+1}^{d}\right)}^{T}W(x_{t+1}^{m}-x_{t+1}^{d})\right]$$

Equation (6) is defined in terms of the expected sum of rewards so as to consider the use of a stochastic control policy, π(p|x;θ), which is a common feature of Reinforcement Learning algorithms. Once the objective function is formalized, the use of Reinforcement Learning proceeds to learn the approximately optimal control policy, $\pi^{*}$. Generally, this policy is parameterized by a nonlinear function, π(θ,⋅). This function provides a mapping from an input (i.e., state variables, $x_{t}^{m},$ at the current time index) to an output (i.e., optimal parameter values, $p_{t}$, at the current time index). In practice, artificial neural networks are often used to parameterize the policy because of their flexibility for approximating continuous functions and their suitability for *end‐to‐end* learning. Parameters in the neural network are learned through maximization of the expected sum of rewards and a policy optimization or actor‐critic algorithm. In this study, we use the “Soft Actor‐Critic” (SAC) algorithm (Haarnoja et al., 2018). Interested readers can read the cited paper for more details on the algorithm.

In addition, to minimize the risk of overfitting the hybrid model, only a minimum set of kinetic parameters should be allowed to vary over time. This is achieved by imposing a condition that if a parameter is considered time‐varying, its estimated deviation (i.e. the deviation between its maximum and minimum value) must be greater than 10%. This is a heuristic, which is based on our experience to keep the framework easy to implement. However, this may be tuned according to the study at hand or implemented systematically by determining the minimum number of time‐varying parameters via sensitivity analysis. Finally, the current work was implemented in Python v3.7.10 using, *SciPy v1.6.2*, *NumPy v10.20.1*, and *PyTorch v1.8.1* libraries. To ensure a reasonable computational cost, the maximum number of training iterations was set as I=10, the maximum training epochs was set as $N_{e,\max}=200$ during each iteration with 500 training trajectories in each training epoch. These are hyperparameters that assign a computational budget to the training process. Additionally, the number of training trajectories has bearing on the accuracy in estimation of the policy gradient – a key consideration in this approach. One training epoch using the SAC algorithm takes 30 s on a MacBook Pro with 16 GB of RAM and a 2.6 GHz Intel i7 processor.

The definition of algorithmic hyperparameters is explicit to implementation of any RL algorithm. In this study, we ensure that our neural network policy is an expressive neural network. In RL practice, there is a tendency to over‐parameterize neural policies with large numbers of hidden neurons. In this case, it was not deemed necessary, however networks are generally thought to become smoother as they become larger, which can be beneficial for gradient‐based approaches to optimization of the network parameters. Additionally, annealing the learning rate of the optimizer (ADAM was used in this study) is known to improve training. Finally, the initialization of neural policy parameters is known to affect the performance of gradient‐based RL methods e.g. reinforce and SAC. In this study, we utilized a ‘Kaiming normal' initialization, which is particularly popular for use in networks with rectified linear unit activation functions ‐ as also used in this study.

## 4 RESULTS AND DISCUSSION

Recall that Scenarios 1 and 2 assume that state variables can be measured once per 2 h, consistent with high‐throughput experimental facilities capable of online measurements (Long et al., 2014; Szita et al., 2005). These two scenarios aim to investigate the performance of the current framework when there is an adequate amount of data. In Scenarios 3, however, sampling frequency was reduced to once per 28 h (6 data points per batch). This aims to test the framework's capability subject to a limited number of data points, as is the case for many large‐scale bioprocesses where data can be only collected once or twice per day to minimize labor cost. However, given the presence of metabolic regulation mechanisms, it is unlikely that kinetic parameters vary dramatically.

For all three scenarios, model fitting performance and parameter estimation results (i.e., the objective score in Equation (6)) were assessed based on the sample average from 500 Monte‐Carlo simulated trajectories within the final training epoch. In addition, the termination criterion for training is defined. The termination criterion has two logic conditions, which require fulfilling for training termination. The first tracks whether there have been three consecutive epochs with no improvement in the sum of the exponentiated negative mean squared errors associated with the state trajectories. This assesses whether training has converged to a region of the neural network's parameter space. The second criterion is that this value should additionally exceed a set score (equal to 2.9). The set score was chosen because the maximum value of the sum of exponentiated negative mean squared errors is 3.0. If both conditions are fulfilled then training is terminated. Clearly, such conditions are not strict and can be defined according to the preference of the implementation.

### 4.1 Results of Scenario 1

In this scenario, the RL policy network includes two hidden layers with 16 neurons in each layer in the first case study, and the Leaky ReLu function is set as the activation function in the hidden layers. The two‐hidden layer structure is deemed sufficient to approximate the optimal policy. In the second case study, as the number of kinetic model parameters (i.e. the dimensionality of the network output) is increased with the addition of the Haldane model, the policy network is designed to include two hidden layers with 32 neurons in each layer. The first and second case study took 3 and 9 iterations to converge, respectively. The reason why the second case study requires more iterations is due to its more complex model structure, which makes the model structure identification and parameter estimation tasks more challenging for RL to learn.

**Figure 2:**
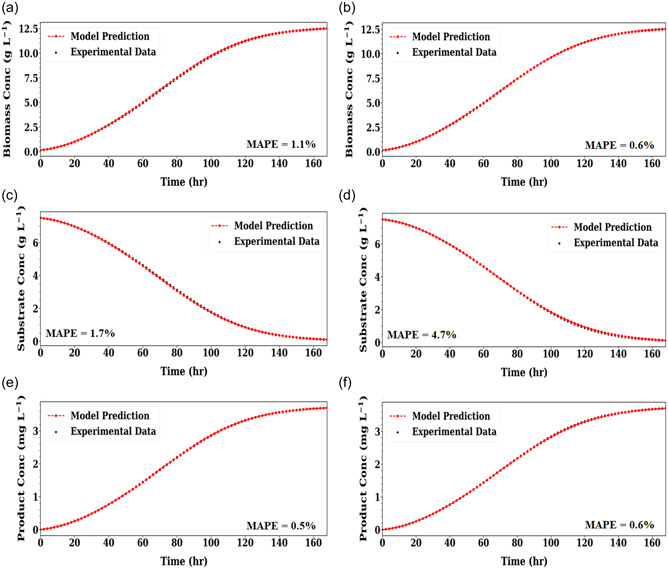
Scenario 1 hybrid model prediction of biomass (a)–(b), substrate (c)–(d) and product concentration (e)–(f) on the in‐silico experimental data set for Case Study 1 (left) and Case Study 2 (right). Mean absolute percentage error (MAPE) between model prediction and experimental data shown.

Figure 2 demonstrates how the hybrid model fitted both case studies well, with the model fitting error (i.e., mean absolute percentage error, MAPE) between the model simulation result and the experimental data generally less than 2%. Not only was the data fitted well, the ground‐truth parameters were accurately recovered, as shown in Table 3, which tabulates the estimated parameter values and their relative error with respect to the ground‐truth values. This shows how current framework effectively captures the dynamics of the combinatorial kinetic system. Moreover, the framework can automatically identify the ‘correct' model structure. Specifically, $\mu_{\mathrm{mh}}$ is correctly estimated to be zero, correctly “switching off” the Haldane model, which is not included in the ground‐truth mechanism.

**Table 3:** Estimated parameter values and relative error between estimated and ground‐truth value of the parameters for Scenario 1 and 2

Scenario 1									
	$\mu_{\mathrm{mm}}$	$\mu_{\mathrm{mc}}$	$\mu_{\mathrm{mh}}$	$K_{\mathrm{sm}}$	$K_{\mathrm{sc}}$	$K_{\mathrm{sh}}$	$Y_{s}$	$Y_{p}$	$K_{i}$
Estimated parameters (CS1)	0.045	0.140	‐	8.08	14.53	‐	0.6	0.3	‐
Relative error (CS1) (%)	0.01	0.03	‐	0.95	3.14	‐	0.26	0.64	‐
Estimated parameters (CS2)	0.043	0.135	5e‐5	7.82	13.23	16.6	0.6	0.3	250
Relative error (CS2) (%)	3.35	3.64	‐	2.25	11.8	‐	0.84	0.19	‐

Scenario 2								
	$\mu_{\mathrm{mm},t}$	$\mu_{\mathrm{mm},t-1}$	$\mu_{\mathrm{mc},t}$	$\mu_{\mathrm{mc},t-1}$	$K_{\mathrm{sm}}$	$K_{\mathrm{sc}}$	$Y_{s}$	$Y_{p}$
Estimated parameters (CS1)	‐	‐	0.1	0.049	‐	4.95	0.8	0.35
Relative error (CS1) (%)	‐	‐	0.78	1.71	‐	0.99	0.48	0.12
Estimated parameters (CS2)	3.2e‐4	1e‐3	0.095	0.05	4.79	5.0	0.8	0.35
Relative error (CS2) (%)	‐	‐	5.21	0.26	‐	0.03	0.37	0.23

Abbreviation: CS, case study.

As stated in Section 3, all the parameters in Scenario 1 are found to be constant as their estimated deviations are lower than 10% as listed in Table S1. It is worth highlighting that, in this study, parameter estimation results were calculated naturally by RL without adding any penalty terms or constraints on possible parameter bounds in the reward function. The use of penalties and constraints may be necessary for more complex case studies; this will be investigated in future work.

### 4.2 Results of Scenario 2

The policy network of this scenario is the same as Scenario 1. It is designed to include two hidden layers with 16 and 32 neurons in each layer in the first and second case study, respectively. The first case study took 3 iterations to converge, while the second case study took 9 iterations.

**Figure 3:**
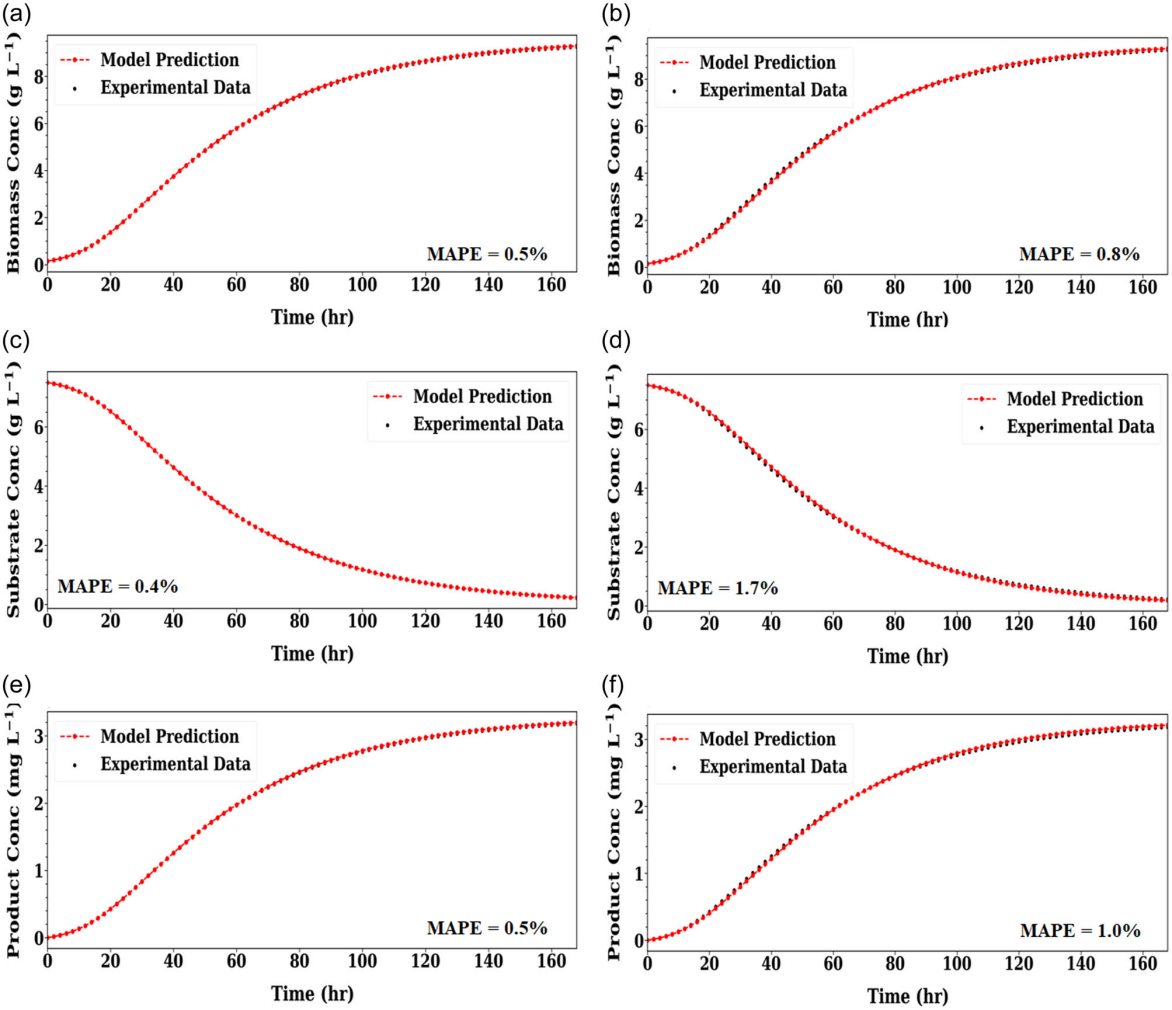
Scenario 2 hybrid model prediction of biomass (a) and (b), substrate (c) and (d) and product concentration (e) and (f) on the in‐silico experimental data set for Case Study 1 (left) and Case Study 2 (right). Mean absolute percentage error (MAPE) between model prediction and experimental data shown.

Model fitting performance and model fitting error for the two case studies are presented in Figure 3. From Table 3, the estimated kinetic parameters are found to be accurate, with a relative error of less than 3% in most cases. In addition, as observed in Table 3, $\mu_{\mathrm{mm},t}$ and $\mu_{\mathrm{mm},t-1}$ were estimated to be zero, in essence, indicating that the Monod model is an incorrect mechanism in the second case study. This demonstrates once again that the current framework can identify the correct model structure for the underlying process. All of the kinetic parameters in this scenario are also concluded to be constant given their negligible estimated temporal deviations as listed in Table S1.

Compared to Scenario 1, Scenario 2 uses the same RL construction but requires more training time to converge. This is because the model structure in the second scenario is more complex than that in the first scenario. In terms of model fitting performance, both scenarios have a fitting error lower than 5% in predictive MAPE. The estimated error of kinetic parameters are also lower than 5%, except $K_{\mathrm{sc}}$ (11.8%) in Case study 2, Scenario 1 and $\mu_{\mathrm{mc},t}$ (5.21%) in Case study 2, Scenario 2. This is mainly caused by the stopping criterion set in this study (i.e. terminating model construction once the reward function reaches 2.9). Although further RL training could reach a higher objective value (since the theoretical maximum is 3.0 when the fitting error is 0), little practical improvement in model fitting was observed in this study. Thus, 2.9 was deemed a good stopping point to balance computational time and model accuracy.

### 4.3 Results of Scenario 3

The policy network of this scenario consists of two hidden layers with 16 neurons in each layer in both case studies. The first case study took 9 iterations to converge, whilst the second case study took four iterations.

**Figure 4:**
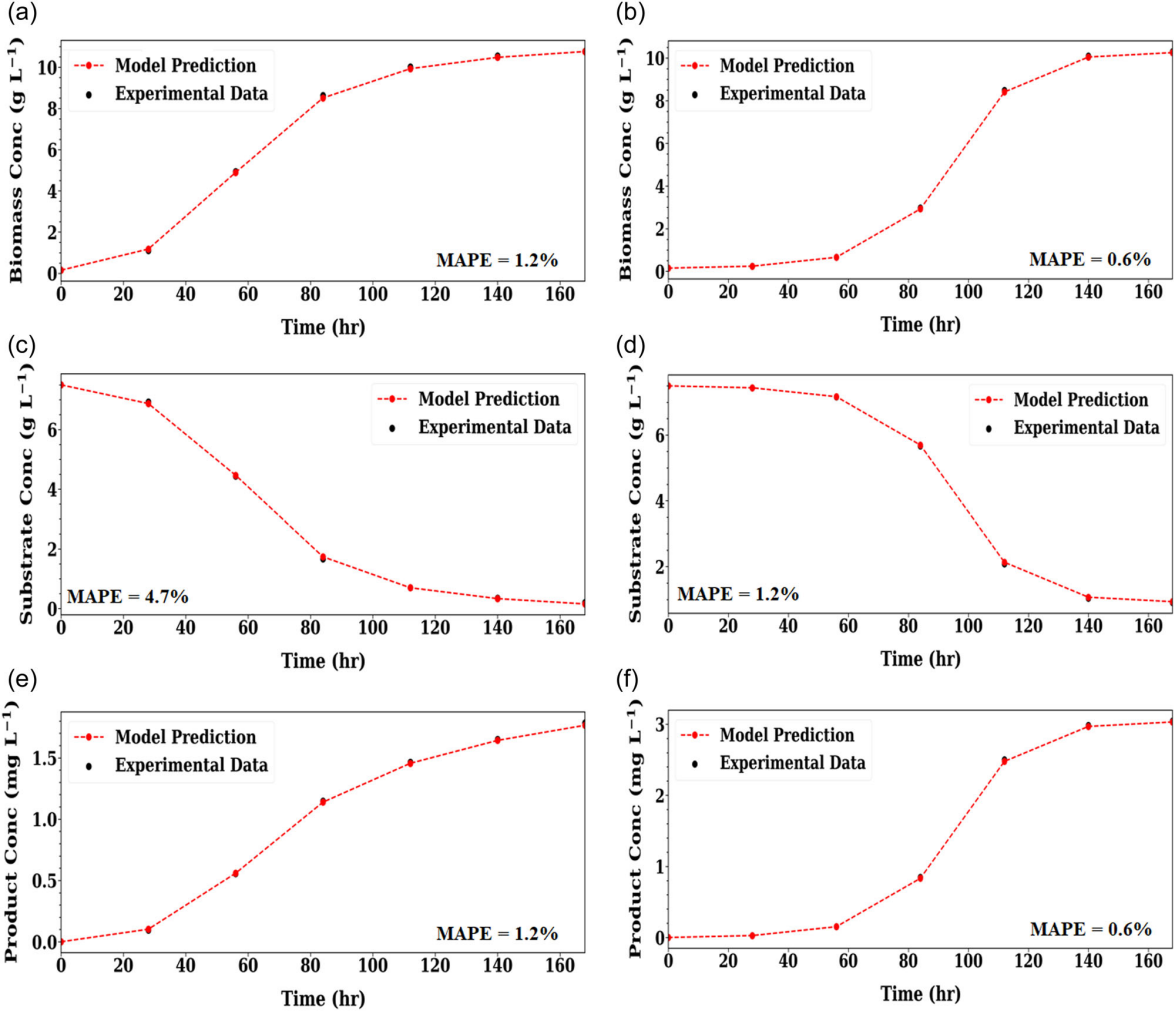
Scenario 3 hybrid model prediction of biomass (a) and (b), substrate (c) and (d) and product concentration (e) and (f) on the in‐silico experimental data set for Case Study 1 (left) and Case Study 2 (right). Mean absolute percentage error (MAPE) between model prediction and experimental data shown.

**Figure 5:**
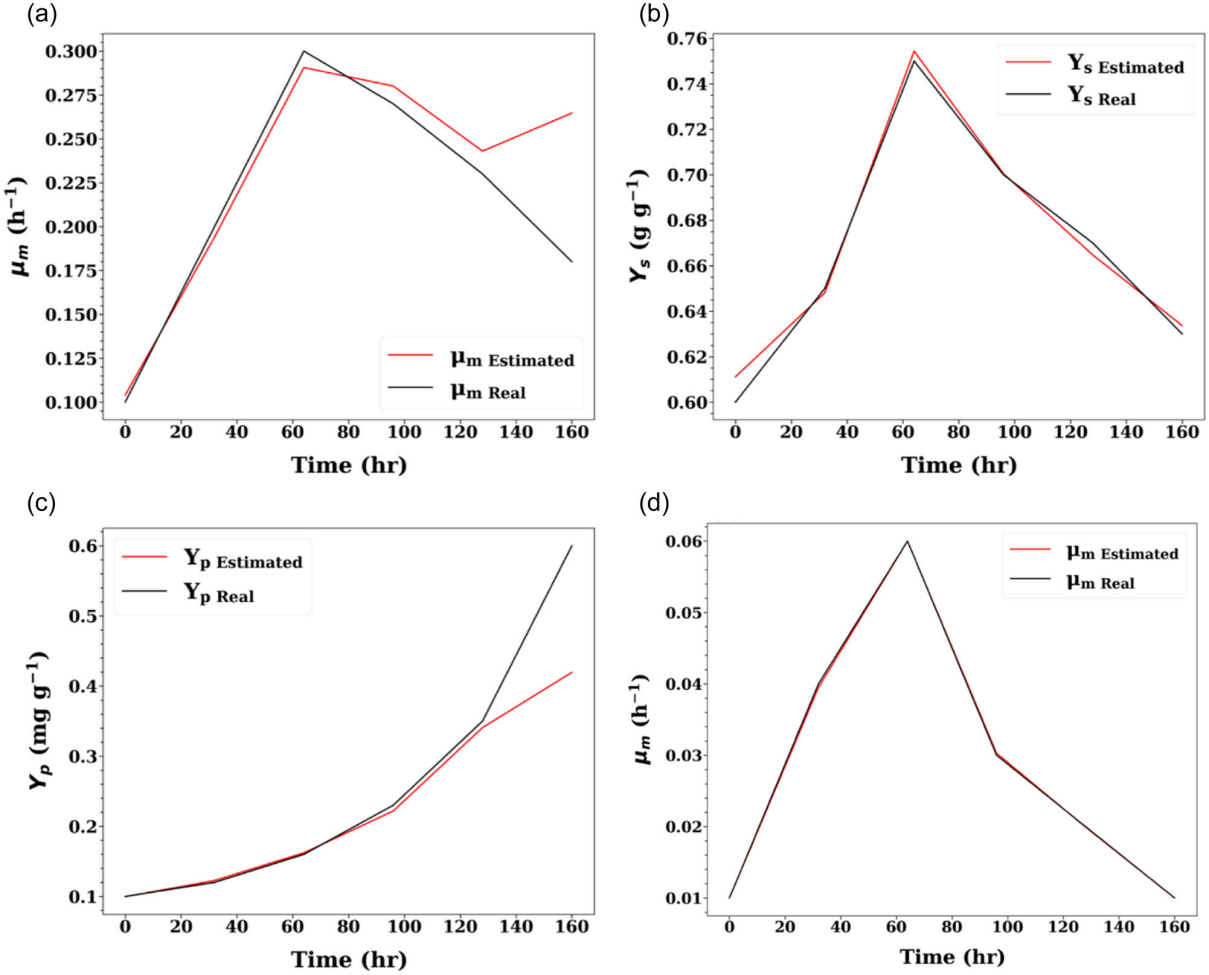
Comparison of Scenario 3 estimated and ground‐truth time‐varying parameter values at each time index for the maximum specific growth rate $\mu_{m}$ (a), substrate yield coefficient $Y_{s}$ (b) and product yield coefficient $Y_{p}$ (c) in Case study 1, and maximum specific growth rate $\mu_{m}$ (d) in Case study 2. Black lines denote the ground‐truth while red lines denote the estimated parameter values. Mean absolute percentage error (MAPE) between model prediction and experimental data shown.

Figure 4 presents the model fitting performance and the model fitting error. It is observed that the constructed hybrid model can simulate the time‐varying kinetic system well, with an error of less than 5%. Table 4 presents the parameter estimation result. From the table, it is found that the framework can accurately pinpoint time‐varying parameters (Case study 1 has 3 time‐varying parameters; Case study 2 has 1 time‐varying parameter) with an estimation error less than 5% in most cases. In addition, Figure 5 presents the estimated dynamic changes of time‐varying parameters of the two case studies. As shown in Figure 5(a)–(c), the parameter fitting result of the first case study is less accurate compared to the second one (Figure 5(d)). However, given the high predictive performance of the model, it is deemed that this discrepancy could correspond to the identification of another local optima. This could be mainly caused by the lack of process information, as more informative data must be provided to account for the higher degrees of freedom in the time‐varying parameter space (i.e., more time‐varying parameters). An alternative approach to further improve the model accuracy is to increase the tolerance objective score (e.g., set this as 2.98 rather than 2.90) and further tune the hyperparameters of the SAC algorithm. However, this could increase the computational time cost and possibly introduce overfitting to the hybrid model.

**Table 4:** Comparison of the time‐varying nature of the estimated parameters and the relative percentage error between estimated and ground‐truth parameter values for Scenario 3

	Kinetic model parameter				Relative error (%)			
	$\mu_{m}$	$K_{s}$	$Y_{s}$	$Y_{p}$	$\mu_{m}$	$K_{s}$	$Y_{s}$	$Y_{p}$
Case study 1								
Ground‐truth	V	C	V	V	11.1	<0.5	0.7	6.7
Estimated	V	C	V	V				
Case study 2								
Ground‐truth	V	C	C	C	0.4	<0.5	<0.5	<0.5
Estimated	V	C	C	C				

*Note*: The notation “V” and “C” indicates each parameter as time‐varying or constant, respectively.

If we now compare the two case studies within Scenario 3, we seen that although the number of kinetic parameters in the two case studies is the same, the training time for the first case study is three times that required for the second case study. We hypothesize this is due to the higher number of time‐varying parameters. As the presence of time‐varying parameters can greatly expand solution space and the degrees of freedom for parameter estimation, it is expected that greater computational cost is needed and the constructed hybrid model may have worse accuracy if there is insufficient data.

### 4.4 The sensitivity of the framework to measurement noise

In this section, we explore the sensitivity of the RL framework to identify ground‐truth parameters despite biomass, substrate and product concentrations being subject to various amounts of measurement noise. Specifically, we re‐examine Scenario 1, Case study 2 with 10%, 20%, 30%, and 40% additive white noise. As before, the ground‐truth parameters are detailed by Table 1. An additional difference between this study and that presented in Section 4.1, is that this time, the parameters are predicted at six discrete points over the 168 h horizon, such that they are allowed to change every 28 h. Hence, in this case we are testing the capabilities of the methodology simultaneously under sparse and noisy data. This is to mimic industrial operations in which data collection is likely to be carried out less frequently than lab‐scale experiments. In addition, due to the rapid development of fermentation process analytical devices, measurement errors for concentrations of biomass, substrates, and product are normally less than 20% even for large‐scale operation. Therefore, the 30% and 40% noise scenarios are mainly studied to test robustness of the proposed framework.

The results of the parameter estimation for the different noise levels are shown in Table 5. In all noise conditions, the methodology identifies the correct model structure, with only parameters from the Monod and Contois models active. The identification of the substrate and product yield coefficients is highly accurate for all noise levels, with the relative error between the ground‐truth and predicted parameters for both 10% and 20% noise levels below 1%. Whereas, the predictions for the Monod and Contois substrate inhibition parameters are subject to relatively larger but consistent inaccuracies for all noise levels of approximately 10% and 20%, respectively. With regard to the growth rates, as the noise level increases the relative accuracies in growth rate predictions change, with higher accuracies in the Monod growth rate observed at high noise levels (and vice versa for the Contois growth rate). However, the average growth rate's error is similar for all noise levels.

**Table 5:** Estimated parameter values, and relative error between estimated and ground‐truth value of the parameters for Scenario 1, Case study 2 for difference percentages of additive white measurement noise

	Kinetic parameters								
	$\mu_{\mathrm{mm}}$	$\mu_{\mathrm{mc}}$	$\mu_{\mathrm{mh}}$	$K_{\mathrm{sm}}$	$K_{\mathrm{sc}}$	$K_{\mathrm{sh}}$	$Y_{s}$	$Y_{p}$	$K_{i}$
Estimated parameters (10%)	0.037	0.13	‐	7.00	12.0	‐	0.60	0.30	‐
Relative error (10%)	17.60	5.56	‐	12.3	20.0	‐	0.31	0.64	‐
Estimated parameters (20%)	0.038	0.127	‐	7.01	12	‐	0.31	0.60	‐
Relative error (20%)	15.9	9.26	‐	12.3	20.0	‐	3.73	0.42	‐
Estimated parameters (30%)	0.046	0.10	‐	7.0	12.0	‐	0.31	0.65	‐
Relative error (30%)	2.22	26.2	‐	12.5	20.0	‐	10.4	8.33	‐
Estimated parameters (40%)	0.042	0.114	‐	7.0	12.0	‐	0.32	0.64	‐
Relative error (40%)	7.74	18.4	‐	20.0	12.23	‐	4.63	7.23	‐

Despite the presence of inaccuracies in the parameters discussed, the framework is able to identify highly accurate predictive models. Figure S1 shows the model predictions and experimental data for noise levels of 20% and 40%, together with the respective mean absolute percentage errors for each component. In both cases, the model regresses the data accurately, with maximum percentage errors of 10.75% in the substrate concentration at 40% additive measurement noise. At 20% noise, the error in the substrate concentration is 8.38%. All other components are predicted at higher accuracies. High predictive accuracies are also observed at 10% and 30% additive noise. At 10% the biomass, substrate, and product concentrations are predicted with 2.38%, 5.72%, and 2.79% mean absolute percentage error. Similarly, for 30% noise the respective errors are 8.67%, 16.8%, and 6.42%. The results discussed here further demonstrate the ability of the framework to handle noisy and sparse datasets as commonly observed in bioprocess systems.

### 4.5 Advantages and limits of the framework

Conventionally, model structure identification is formulated as a mixed‐integer nonlinear programming problem (MINLP) to find a minimum number of active terms and simultaneously estimate parameters (Rodriguez‐Fernandez et al., 2006, 2013; Zhang, Savage, Cho & Rio‐Chanona, 2020). Compared to this approach, the proposed framework does not require binary variables. This means no manually tuned penalty term is needed to minimize the number of active binary variables. However, as RL is time‐consuming to train, if the underlying process is not complex (i.e., the process model is not highly nonlinear) and there are no history‐dependent or time‐varying dynamics, the MINLP approach would be more time‐efficient than the proposed framework. Nonetheless, as the solution quality of an MINLP problem is greatly influenced by its model structure, if history‐dependent behavior exists or the developed kinetic model structure is highly nonlinear, the MINLP approach will likely not converge. Thus, the currently proposed framework will be superior in solution quality and computational cost. Moreover, if there are time‐varying parameters present in the model, using MINLP to solve the underlying problem could be particularly challenging or infeasible. Therefore, in such cases, the RL based hybrid model construction strategy would be more efficient and effective.

In addition, compared to the traditional hybrid model construction framework based on the sensitivity equations and the incremental approach proposed by Oliveira (2004); Rodriguez‐Fernandez et al. (2006), the RL‐based model construction approach does not require solving the sensitivity equations, which has practical benefits if the kinetic model structure is either highly nonlinear or non‐smooth. Furthermore, it is unknown if the traditional approach can deal with history‐dependent kinetics as numerical issues may be introduced when solving the sensitivity equations. So far, no research has applied this approach to construct a history‐dependent hybrid model. However, RL is known to be sensitive to implementation and hyperparameter selection. Therefore, the development of more efficient and stable RL algorithms should be explored. Similarly, if a large number of possible model structures are proposed at once for model structure identification (i.e., a large number of kinetic parameters), the RL‐based hybrid model construction framework may have difficulty in training due to the increased complexity of the policy network and unbalanced model input and output dimensions. As a result, more advanced deep RL training algorithms must be employed for these scenarios.

In addition, it should be noted that the use of the model construction framework proposed here does not guarantee identification of a high‐performance model at once (within one model construction iteration). If the constructed model does not satisfy the required accuracy of the case study (e.g., if the contained kinetic model structures significantly deviate from the ground‐truth model, causing large model‐process mismatch), one can always augment more possible model structures and continue model development through the current hybrid modeling framework (i.e., carrying out more iterations) until the requirement is met. However, this is a generic issue applied to all model construction frameworks, thus not specific to the proposed RL‐based method.

One of the major benefits of hybrid modeling approaches is their ability to integrate the flexibility of data‐driven functions and retain a mechanistic structure. More recently, physics‐informed neural networks (PINNs) have shown good application to identification of nonlinear systems (Cai et al., 2021). The idea of PINNs is to leverage the flexibility of machine learning approaches, but regularize them toward satisfying mechanistic expressions. This has proved a powerful approach to identifying higher‐order ordinary differential equations as well as partial differential equations (PDEs), even using sparse and noisy data (Both et al., 2021). The major difference between this study and PINNs is that the predictions generated here explicitly satisfy a mechanistic expression, instead of simply being regularized towards them. In addition, PINNs have not been applied to time‐varying systems before, therefore their performance in these cases is yet to be explored. However, the RL approach relies on the underlying expressions being cheap to simulate. This likely makes it less suited to identifying PDEs than PINNs. In future work, we will look to compare the performance of PINNs and the work proposed.

## 5 CONCLUSION

In this study, we have outlined a Reinforcement Learning based framework for synchronous and automatic kinetic (hybrid) model structure identification and parameter estimation. By reformulating kinetic model parameter estimation as a control problem, the framework identifies a control policy, which automatically infers kinetic model parameters and structure as a function of system state (i.e., biomass, substrate, and product concentration) as the process evolves through time. We test this framework against several case studies on processes that exhibit challenging combinatorial (i.e., Monod, Contois, and Haldane), history‐dependent or time‐varying kinetic behavior. The latter two pose a significant issue for existing optimization‐based frameworks. The RL approach correctly recovered the underlying ground‐truth kinetic model and demonstrated high accuracy in state prediction, observing an average mean absolute percentage error of 1.3% between the predicted and measured system states and a maximum error of 4.7%. This reflects the quality of the approach for kinetic model structure identification and parameter estimation.

The framework possesses further advantages for handling highly nonlinear and nonsmooth kinetic model structures, which pose a challenge to conventional MINLP‐based model structure identification and sensitivity equations‐based hybrid model construction approaches. However, limitations to the proposed RL approach may arise if the model structure is significantly over‐parameterized by a large number of complicated combinatorial kinetic models or many time‐varying parameters. In addition, further research should also explore the application of this framework to cybernetic modeling of bioprocess systems (Aboulmouna et al., 2020), as well as how to quantify the uncertainty of RL‐based hybrid model predictions as this is critical for bioprocess dynamic optimization and control.

## AUTHOR CONTRIBUTIONS

Max R. Mowbray contributes to the paper drafting (lead) and revision (lead), Chufan Wu and Alexander W. Rogers contribute to paper drafting (supportive) and revision (supportive), Ehecatl Antonio Del Rio‐Chanona contrbutes to paper drafting (supportive) and supervision (supportive), Dongda Zhang contributes to paper drafting (supportive), revision (supportive) and supervision (lead).

## CONFLICTS OF INTEREST

The authors declare no conflicts of interest.

## Supporting information

Supporting information.

## Data Availability

Data sharing not applicable to this article as no datasets were generated or analyzed during the current study.
