# Supplementary material for: A reinforcement learning‐based hybrid modeling framework for bioprocess kinetics identification
Source: Biotechnol Bioeng. 2022 Oct 26;120(1):154–68. doi: 10.1002/bit.28262 (PMC10092184; doi:10.1002/bit.28262)
Supplement: Supplementary file 1 — Supporting information. [file BIT-120-154-s001.docx]

# Supplementary material: A reinforcement learning based hybrid modelling framework for bioprocess kinetics identification

Max R. Mowbray^1^, Chufan Wu^1^, Alexander W. Rogers^1^, Ehecatl Antonio Del Rio-Chanona^2^, Dongda Zhang^1,*^

1: Centre for Process Integration, Department of Chemical Engineering and Analytical Science, University of Manchester, Manchester, UK

2: Centre for Process Systems Engineering, Imperial College London, South Kensington Campus, London, UK

*: Corresponding authors, email: [dongda.zhang@manchester.ac.uk](about:blank) (Dongda Zhang).

**Supplementary**

Here, we present supporting information regarding the variation of estimated kinetic parameters for Scenarios 1 and 2, and the respective case studies. The results are detailed by Table S1.

Table S1: Deviation of estimated parameters over the batch time for Scenario 1 and 2.

| Variation (Prediction deviation) (%) | | | | | | | | | | | | | | | | |
| --- | --- | --- | --- | --- | --- | --- | --- | --- | --- | --- | --- | --- | --- | --- | --- | --- |
| Scenario 1 | | | | | | | | | | | | | | | | |
|  | $\mu_{mm}$ | $\mu_{mc}$ | | $\mu_{mh}$ | | $K_{sm}$ | | $K_{sc}$ | | $K_{sh}$ | | $Y_{s}$ | | $Y_{p}$ | | $K_{i}$ |
| Case Study 1 | 0.12 | 0.11 | | - | | 0.06 | | 0.11 | | - | | 0.41 | | 1.9 | | - |
| Case Study 2 | 0.82 | 0.37 | | 1.9 | | 055 | | 0.49 | | 1.2 | | 0.47 | | 1.3 | | 8.9 |
| Scenario 2 | | | | | | | | | | | | | | | | |
|  | $\mu_{mm,t}$ | | $\mu_{mm,t-1}$ | | $\mu_{mc,t}$ | | $\mu_{mc,t-1}$ | | $K_{sm}$ | | $K_{sc}$ | | $Y_{s}$ | | $Y_{p}$ | |
| Case Study 1 | - | | - | | 0.79 | | 0.41 | | - | | 1.2 | | 0.25 | | 0.4 | |
| Case Study 2 | 8.5 | | 9.6 | | 1.6 | | 0.2 | | 0.42 | | 0.1 | | 0.75 | | 0.45 | |

Here, we present visualisation of the model fit for 20% and 40% additive white Gaussian noise, based on discussion in Section 4.4.


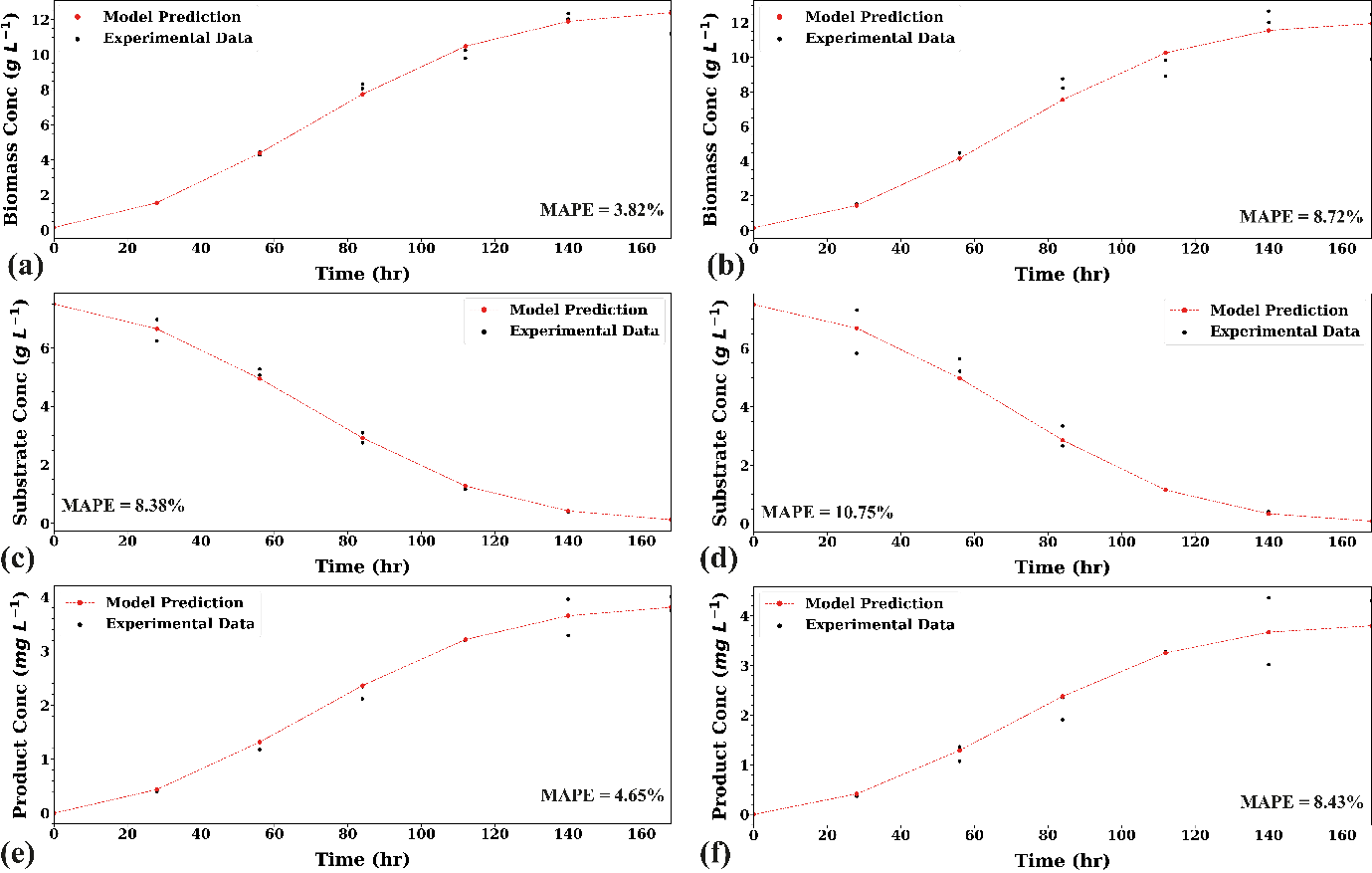


Figure S1: Scenario 1, Case Study 2 hybrid model prediction of biomass (a)-(b), substrate (c)-(d) and product concentration (e)-(f) on the in-silico experimental dataset for 20% (left) and 40% (right) additive white measurement noise. Mean absolute percentage error (MAPE) between model prediction and experimental data shown.
